# Flap monitoring with incisional negative pressure wound therapy (NPWT) in diabetic foot patients

**DOI:** 10.1038/s41598-022-20088-9

**Published:** 2022-09-20

**Authors:** Jun Ho Park, Ji-Ung Park

**Affiliations:** Department of Plastic and Reconstructive Surgery, SMG-SNU Boramae Medical Center, Seoul National University College of Medicine, 20 Boramae-ro 5-gil, Dongjak-gu, Seoul, 07061 Republic of Korea

**Keywords:** Diseases, Health occupations, Medical research

## Abstract

Various types of flaps are considered as reconstructive options for patients with diabetic foot ulcer. However, flap reconstruction for diabetic foot ulcer treatment is particularly challenging because of the relatively limited collateral perfusion in the distal lower extremity. This study evaluated the efficacy and safety of a novel postoperative monitoring procedure implemented in conjunction with negative pressure wound therapy immediately after flap operations for treating diabetic foot. A retrospective analysis was performed on diabetic foot patients who underwent free flaps and perforator flaps from March 2019 through August 2021. The surgical outcomes of interest were the rates of survival and complications. On the third postoperative day, patients underwent computed tomography angiography to check for pedicle compression or fluid collection in the sub-flap plane. Monitoring time, as well as comparisons between NPWT and conventional methods, were analyzed. Statistical analysis was performed between the two groups. This study included 26 patients. Among patients, the negative pressure wound Therapy treated group included 14 flaps and the conventional monitoring group included 12 flaps. There was no significant intergroup difference in flap survival rate (*p* = 0.83). In addition, there was no significant intergroup difference in the diameters of perforators or anastomosed vessels before and after negative pressure wound therapy (*p* = 0.97). Compared with conventional monitoring, flap monitoring with incisional negative pressure wound therapy was associated with a significantly lower mean monitoring time per flap up to postoperative day 5. Although conventional monitoring is widely recommended, especially for diabetic foot ulcer management, the novel incisional negative pressure wound therapy investigated in this study enabled effortless serial flap monitoring without increasing complication risks. The novel flap monitoring technique is efficient and safe for diabetic foot patients and is a promising candidate for future recognition as the gold standard for flap monitoring.

## Introduction

One-third of people with diabetes mellitus will develop at least one diabetic foot ulcer (DFU) over the course of the disease, and 5-year mortality rates associated with DFU reach high estimates of even 30%^1^. DFUs are among the most challenging complications to manage, and treatment failure is a relatively common outcome of the limited treatment options. Flap reconstruction for DFU treatment is particularly challenging because of the relatively limited collateral perfusion in the distal lower extremity^2–5^.

Negative pressure wound therapy (NPWT) has emerged as an encouraging option for wound management, particularly for wounds of the extremities, including defects resulting from diabetic gangrene, trauma, and malignancies. NPWT works by facilitating improved blood flow, wound contraction, and interstitial fluid removal^6,7^. In recent decades, the indications for NPWT have included incisional wound management, as well as its use as an adjunctive component of skin grafting and flap salvage procedures (to effectively improve graft take)^8–11^.

Even though NPWT has been widely implemented with favorable outcomes, controversy remains regarding its safe applicability to flaps raised for diabetic foot management. Of particular relevance to this debate is the extensive, protracted postoperative monitoring required after flap surgery, which has been associated with a high risk of infection^12,13^.

The safety concerns over NPWT implementation for free or perforator flaps are reinforced by the scarcity of published studies investigating the efficacy of immediate postoperative NPWT for such flaps. Postoperatively, many surgeons are hesitant to immediately apply NPWT for DFU flaps. Another concern is the potential effect of NPWT on fragile calcified pedicles.

This study evaluated the efficacy and safety of a novel postoperative monitoring procedure implemented in conjunction with NPWT immediately after flap operations for treating diabetic foot. Specifically, compared with conventional monitoring, the novel monitoring system was developed to optimize efficacy and minimize the risk of infection.

## Methods

Our institutional review board (IRB No. 2020–10-150,478) approved this retrospective review of postoperative DFU patients monitored using our novel immediate postoperative monitoring protocol in conjunction with NPWT. All patients included in this analysis provided written informed consent after 1 postoperative month at the outpatient clinic. The study was conducted in accordance with the Declaration of Helsinki and its later amendments. Informed consent was obtained from patients for all surgical procedures and wound management, and for the possible use of anonymized photographs. Eligible patients were those who underwent flap procedures (free flap, perforator flap) for DFU treatment from March 2019 through August 2021. This was a single-center study, and a single attending plastic surgeon (J.H. Park) with 17 years of experience performed all procedures, which were carried out using standard methods.

**Figure 1:**
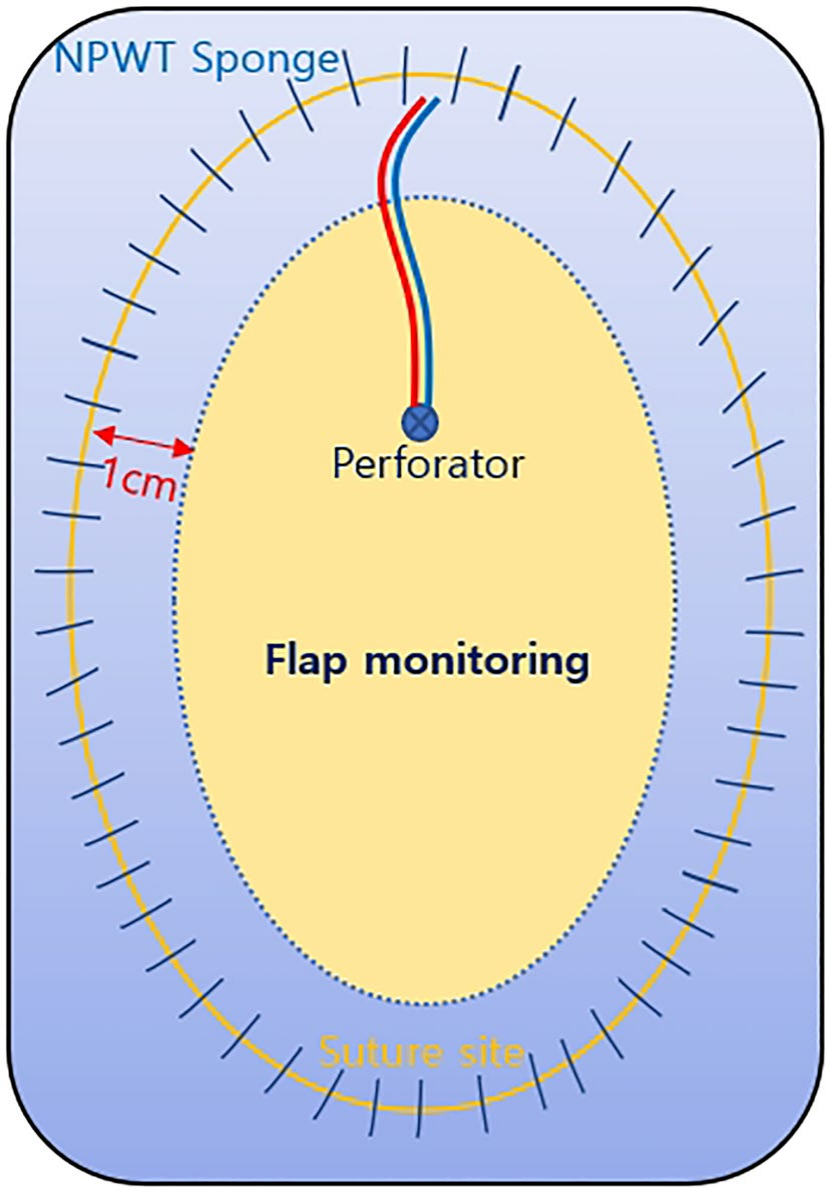
Schematic diagram of the novel NPWT system for postoperative flap management.

After flap inset, antibiotic ointment–coated Physiotulle (hydrocolloid-based, non-adherent wound contact layer, Coloplast, Ltd. Peterborough, UK) was used to fully protect the suture site, over which a black NPWT sponge (V.A.C. Granufoam, KCI, now part of 3 M company, San Antonio, TX, USA) was then placed. The device (INFOV.A.C. Therapy Unit, KCI, now part of 3 M company, San Antonio, TX, USA) was set to − 75 mmHg on continuous mode. To facilitate serial flap monitoring, a large window—consisting of almost the entire flap—was routinely formed because the sponge margin did not cross the flap paddle more than 1 cm from the incision site (Fig. 1). For all flaps, monitoring was conducted by serial examinations through the transparent window created by as part of the novel NPWT technique. NPWT apparatuses were removed after 5 postoperative days.

Some patients were categorized into a conventional monitoring group. These patients underwent standard manual dressing, along with manual monitoring carried out by a provider who used sterile surgical gloves while cleaning the suture site with saline-soaked gauze. Sterile gauze was used to loosely cover the flap’s suture site after the application of antibiotic-coated Physiotulle.

Immediately postoperatively, the size of the flap surface was measured using standard planimetry method. all flaps were monitored closely by a single physician in department of plastic and reconstructive surgery. The 4-hourly monitoring, which involved observation of flap color, temperature, capillary refill, and external Doppler ultrasonography was performed. After 24 postoperative hours, flap monitoring was conducted every 8 h for the next 24 h. Thereafter, monitoring was conducted every 12 h until the fifth postoperative day. Postoperatively, all patients remained hospitalized until the senior surgeon deemed them medically and surgically suitable for discharge. The surgical outcomes of interest were the rates of survival and complications (including infection, seroma, hematoma, and flap necrosis).

**Figure 2:**
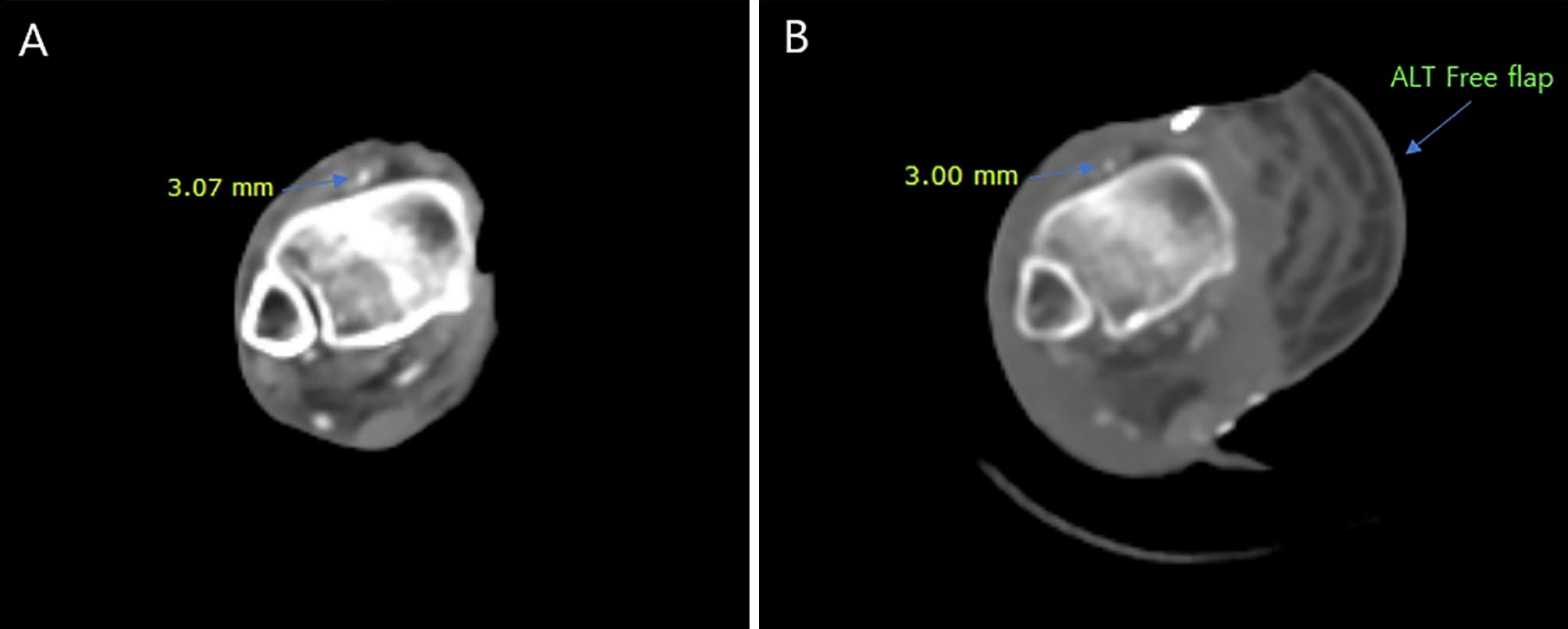
Computed Tomography (CT) angiography finding. Patent flap pedicle was confirmed without the evidence of compression. The diameter of each pedicle was measured. (**A**) Before NPWT system apply, DD* = 3.07 mm (**B**) After NPWT system apply, DD = 3.00 mm. DD* = Diameter of Dorsalis pedis artery

On the third postoperative day, patients underwent computed tomography (CT) angiography to check for pedicle compression or fluid collection in the sub-flap plane (Fig. 2). Pedicle diameter (before and after NPWT) was determined via visualization with a picture archiving and communication system. For each patient, the total monitoring time was recorded for 5 postoperative days, and surgical outcomes were evaluated after 1 postoperative month at the outpatient clinic. The normality test was performed using Kolmogorov–Smirnov test was performed. Monitoring time, as well as comparisons between NPWT and conventional methods, were analyzed using the Mann–Whitney U test. Statistical analyses were conducted using SPSS Statistics for Windows, version 26.0 (IBM Corp., Armonk, NY, USA). Statistical significance was defined by *p* values < 0.05.

## Results

This study included 26 patients with a mean age of 55 years (range: 44–88 years). The mean follow-up duration was 6.2 months. Table 1 summarizes all other characteristics. Among DFU patients, the NPWT group included 14 flaps (six free flaps and eight perforator flaps), and the conventional monitoring group included 12 flaps (five free flaps and seven perforator flaps). The mean flap surface area was 88.6 cm^2^, and the mean operation time was 296.5 min (284.9 min in the NPWT group vs. 303.8 min in the conventional group; this difference was not statistically significant). All flaps were anterolateral thigh flaps.

**Table 1:** Demographic findings of the patients.

Criteria	Conventional group	NPWT group	*P*-value
Patient numbers	12	14	
Age, y (range)	53.8 (45–88)	55.6 (44–84)	0.84
Body mass index, kg/m^2^ (range)	25.9 (17–32)	24.7 (20.8–28.9)	0.71
**Type of flaps**			0.53
Free flap	5	6	
Perforator flap	7	8	
**Hypertension**			0.64
Yes	5	5	
No	7	9	
**Smoking**			0.61
Yes	6	8	
No	6	6	
**Cardiovascular disease**			0.82
Yes	4	3	
No	8	11	

NPWT: negative pressure wound therapy.

There was no significant intergroup difference in flap survival rate (92.9% in the NPWT group vs. 91.7% in the conventional group, *p* = 0.83) (Table 2). In addition, there was no significant intergroup difference in the diameters of perforators or anastomosed vessels before and after NPWT (2.3 mm before NPWT application vs. 2.4 mm after NPWT application, *p* = 0.97).

**Table 2:** Surgical outcomes of conventional monitoring and NPWT monitoring groups.

	Conventional group	NPWT group	*P*-value
Total number of flaps	12	14	
Mean flap size (cm^2^)	87.8 ± 49.1	94.2 ± 41.7	0.45
Mean operation time (min)	284.9 ± 65.7	303.8 ± 38.1	0.61
Flap survival	11	13	0.83
Total Monitoring time*(min)	86.4	225.1	
Flap complication	3	1	
Infection	2	–	
Hematoma	–	–	
Seroma	–	–	
Partial necrosis	–	–	
Complete necrosis	1	1	

NPWT: negative pressure wound therapy.

*Total monitoring time: up to postoperative day 5.

There were no instances of abnormality of monitoring (flap color, temperature, capillary refill and doppler sound) in both groups. Postoperative complications, such as hematoma or seroma formation, or wound dehiscence were not identified. However, one flap in the NPWT group sustained complete necrosis after suffering from venous congestion, which was successfully managed with 2 months of serial debridement and split thickness skin graft. One free flap in the conventional monitoring group sustained complete flap necrosis after signs of venous congestion; this was treated with an alternative peroneal artery perforator-based flap. Two patients in the conventional monitoring group were treated for surgical site infection after purulent discharge was observed.

Compared with conventional monitoring, the novel NPWT monitoring system was associated with a significantly lower mean monitoring time per flap up to postoperative day 5 (86.4 min in the NPWT group vs. 225.1 min in the conventional group, *p* < 0.05).

### Case #1

**Figure 3:**
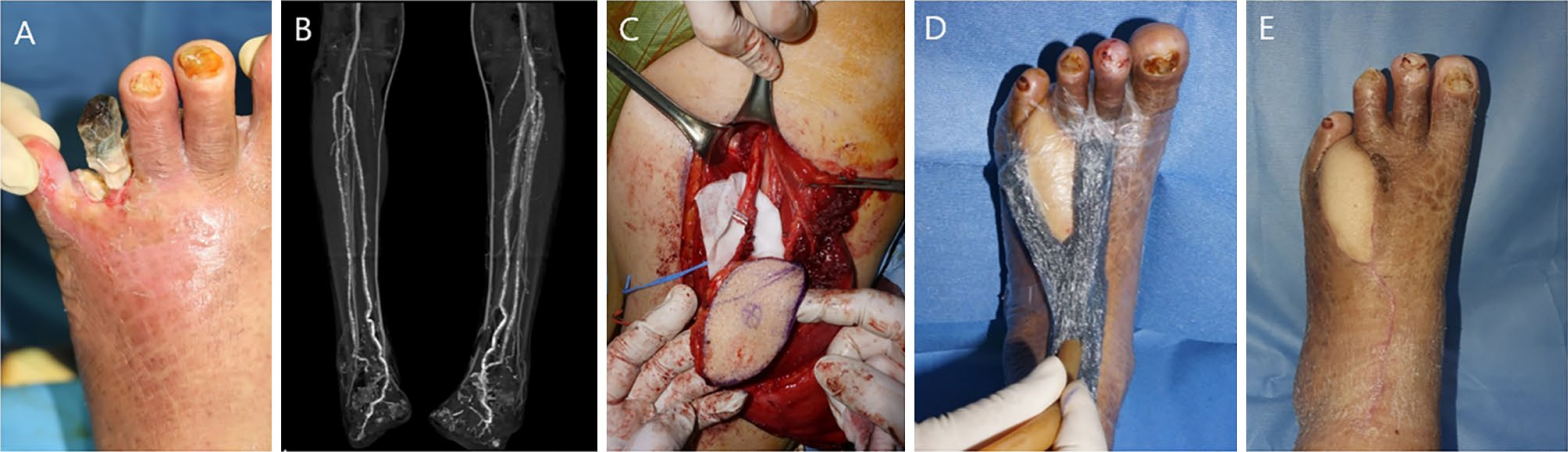
The first case of NPWT monitoring system in diabetic foot patient. (**A**) Photographic findings of 57-year-old male patient who was diagnosed with diabetic gangrene on left foot (**B**) CT angiography finding of lower extremities (**C**) Elevation of ALT free flap (**D**) NPWT was applied immediately after operation (E) Postoperative photographic finding; 3 months.

A 57-year-old man with diabetes mellitus presented with a painful, raw superficial lesion on his left foot. A free ALT flap transfer was performed under general anesthesia to resurface the wound. The designed flap was 5 cm × 10 cm for a 2 cm × 8 cm defect. The dorsalis pedis artery and veins were used to perform flap inset and vessel anastomosis, and the direct closure method was used to close the donor site. The procedure took 230 min and was completed by covering the flap suture site margins with a contact barrier (Physiotulle) underlying a sponge from the NPWT monitoring system (Fig. 3). The entire flap region was covered with thin transparent film and NPWT device was set to − 75 mmHg on continuous mode. CT angiography was performed on the third postoperative day to confirm an intact pedicle. The NPWT monitoring system was changed on the fifth postoperative day. Flap survival was confirmed at the 3-month follow-up visit, and no complications had occurred up to that point.

### Case #2

**Figure 4:**
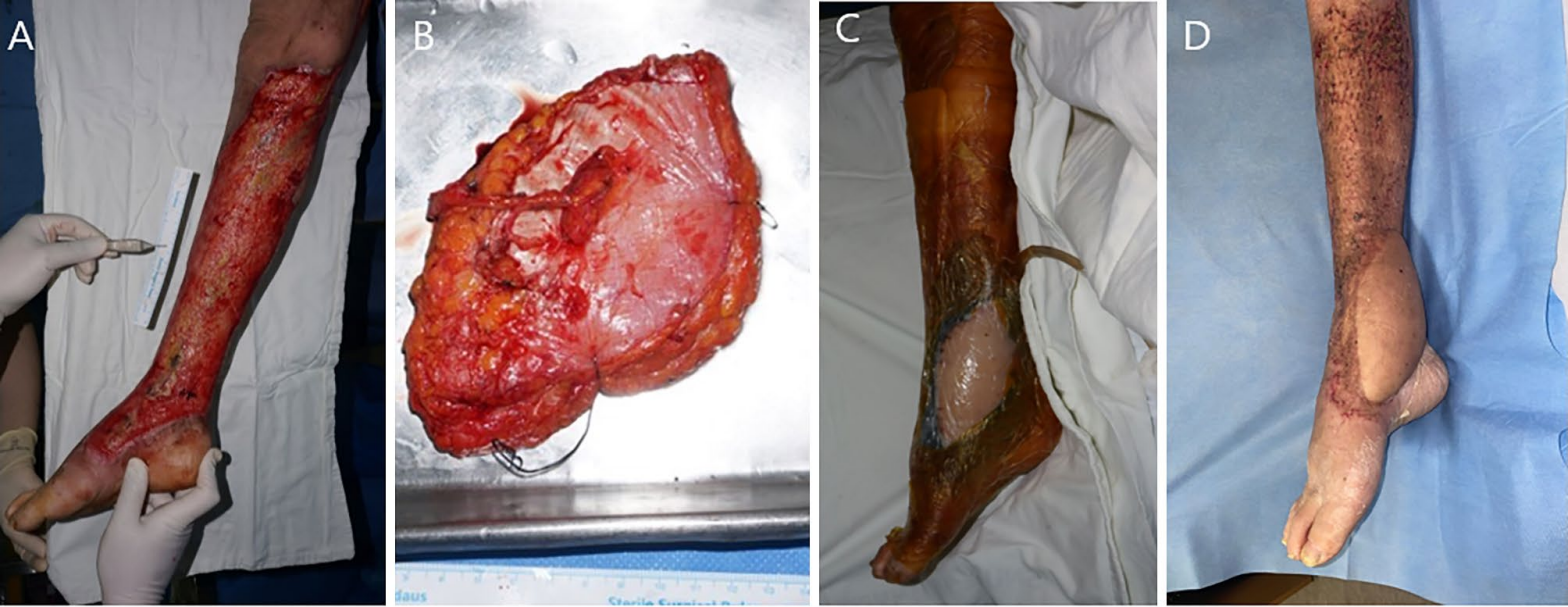
The second case of NPWT monitoring system in diabetic foot patient accompanied with necrotizing fasciitis. (**A**) Photographic findings of 74-year-old female patient who was diagnosed with diabetic foot accompanied with necrotizing fasciitis on right lower extremity (**B**) Harvested ALT free flap (**C**) Immediate postoperative photographic finding (**D**) Postoperative photographic finding; 7 months.

A 74-year-old woman was admitted with a painful, raw surface lesion on her right lower extremity. Under general anesthesia, the patient underwent free ALT flap transfer with a meshed split-thickness skin graft to cover the defect, which was 54 cm × 12 cm. The designed flap was 15 cm × 9 cm. The dorsalis pedis artery and veins were used to perform flap inset and vessel anastomosis, and the direct closure method was used to close the donor site. The procedure took 387 min and was completed by covering the flap suture site margins with a contact barrier (Physiotulle) underlying a sponge from the NPWT monitoring system (Fig. 4). The entire flap region was covered with thin transparent film and NPWT device was set to − 75 mmHg on continuous mode. CT angiography was performed on the third postoperative day to evaluate the pedicle status. The NPWT monitoring system was changed on the fifth postoperative day. Flap survival was confirmed at the 6-month follow-up visit, and no complications had occurred up to that point.

## Discussion

DFUs are a major cause of hospitalization among individuals with diabetes. Recently, 5-year survival rates associated with DFUs have increased, and amputation rates have decreased due to improvements in diabetes treatment and the development of effective treatment guidelines^14–17^. Additionally, increased knowledge about the pathophysiology of DFU formation has facilitated the development of various medical and surgical options to promote wound healing ^18,19^. Among the various emergent treatment options, the roles of NPWT cover the period from the management of the preoperative open wound to that of the postoperative flap site.

NPWT is a non-invasive intervention that promotes wound healing by removing the fluid from the wound through a sealed sponge connected to a collection canister using a vacuum device with controlled negative pressure. Argenta and Morykwas introduced the technique, and NPWT achieves its outcomes via the following elements: wound transudate removal, infection prevention, blood flow optimization, and edema minimization^20–24^. Additionally, with NPWT for open wounds, mechanical deformation plays a key role in tissue expansion^25^. Hence, NPWT has been implemented for a wide range of specific clinical indications, including promoting wound healing and minimizing complications at incision sites^26,27^. Particularly in the contexts of unstable or high-tension wounds, the negative pressure generated by vacuum devices enhances tissue perfusion and removes fluid and infectious components from wounds^28,29^. The results of this study suggest that there may be effects such as fluid removal along with an increase in tissue perfusion to achieve viability of the flaps.

Flap operations are among the most important reconstructive surgical procedures, especially for wounds involving exposed bone. Successful NPWT implementation after flap coverage is becoming progressively widespread for both traumatic and incisional wounds, and the technique has emerged as an efficient alternative to traditional flap salvage techniques, such as leech therapy and heparin injection^8–11^. NPWT has proven its efficacy for removing excess interstitial fluid from beneath flaps and for facilitating the resolution of venous congestion to promote flap viability^10,11,30^. The efficacy of NPWT for minimizing complication rates and improving salvage rates associated with pedicled flaps and free muscle flaps has been demonstrated, including in comparative terms relative to traditional flap management^11,31–33^. However, no publications have reported on NPWT implementation immediately after flap reconstruction for amphibolic DFU patients.

Bi et al.^34^ introduced a modified NPWT technique, involving standard placement of the VAC sponge with a pressure of − 125 mmHg, for a skin-containing free flap. This technique enabled serial flap examination by creating a small window in the distal end of the flap. However, the authors were not able to visually monitor the entire flap; therefore, they could not address the issue of NPWT safety for free flaps. Lin et al.^35^ compared NPWT (immediately after head and neck reconstruction using free flaps) with conventional wound care and found NPWT to be associated with lower complication and infection rates (9.7% vs. 37% and 0.0% vs. 14.8%, respectively). However, because of full coverage of the flap by the VAC sponge, visual and Doppler monitoring were not possible. Chen et al.^36^ immediately applied NPWT following lower extremity flap reconstruction; however, their study did not confirm the safety of direct NPWT application on flap pedicles and also the free flaps were not included.

The novel NPWT technique used in this study was developed to monitor entire flaps and address safety concerns. Especially in diabetic foot wounds, excessive pressure may injure soft tissue including vascular structure. This is why the pressure was set to − 75 mmHg referring to the fact that other previous studies set the pressure from − 75 mmHg to − 125 mmHg to ensure the safety of the flap. Flap color changes, capillary refilling status, and flap warmth can be assessed through a transparent film covering most of the flap area. Interstitial fluid removal through the sponge can proceed with no effect on flap perfusion. Moreover, a newly reported benefit is that the uncertainty over pressure-induced flap pedicle disruption (by the VAC sponge) can be eliminated by CT angiography.

A major consideration of postoperative DFU management after flap surgery is sustainable flap monitoring. Postoperative flap management is crucial for minimizing major complications. Flap maintenance relies heavily on early detection of flap problems^37^. To date, none of the various flap monitoring methods, such as near-infrared spectroscopy, implantable Doppler, and laser Doppler flowmetry, are recognized as the diagnostic gold standard.

The first 24 h after free flap procedures are crucial for monitoring, detecting, and (planning for) managing complications^38,39^. Frequent flap monitoring is regarded as ideal for flap salvage^40^, but the requisite short monitoring intervals and time-consuming steps involved in conventional postoperative flap monitoring are burdensome. Moreover, flaps are susceptible to infection and vascular injury, which can lead to hematoma and seroma formation^41^. Notably, our novel monitoring technique was associated with significantly reduced person-time requirements for postoperative flap monitoring (*p* < 0.05); it was also associated with a lower infection rate. Even when soft tissue inflammation did not completely resolve, transferred fresh flaps tended to improve the adjacent tissue environment and the C-reactive protein level over time. Changing the NPWT sponge on the fifth postoperative day was associated with a slight increase in monitoring time; however, the person-time requirement associated with NPWT group was still less than that associated with conventional monitoring (*p* < 0.05).

This study had a few limitations. First, the exact pressure map associated with NPWT was not delineated. However, the NPWT and conventional monitoring groups did not have significantly different flap failure rates from one another, and CT angiography revealed no significant pressure effect by NPWT on flap pedicles. The NPWT-generated pressure was considered minimal. Second, this retrospective study involved relatively short follow-up. Surgical characteristics, such as initial wound condition and flap types, were not evaluated, and the patients were heterogeneous in terms of medical history and flap types, which could have affected surgical outcomes, including complication and survival rates. Furthermore, there were no specific indications or criteria for selecting between conventional monitoring and the NPWT monitoring system. The novel NPWT monitoring system achieved acceptable surgical outcomes compared with those reported in previous studies of DFU patients, and the study findings support the safety of the system. However, due to small sample size and the power analysis was absent, future larger-scale, prospective, and controlled investigations are warranted to validate the findings of this study, which demonstrated the simplicity and safety of a novel NPWT monitoring system in terms of comparisons with conventional manual monitoring.

## Conclusions

Postoperative flap monitoring is vital for flap success. Although conventional monitoring is widely recommended, especially for DFU management, the novel NPWT monitoring system investigated in this study enabled effortless serial flap monitoring without increasing complication risks. The novel flap monitoring technique is efficient and safe for DFU patients and is a promising candidate for future recognition as the gold standard for flap monitoring.

## Supplementary Information

Supplementary Information.

## Data Availability

Availability of Data and Materials. All data generated or analyzed during this study are included in this published article (its supplementary information file).

## Abbreviations

DFU: Diabetic foot ulcer
NPWT: Negative pressure wound therapy
CT: Computed tomography
Hs-CRP: High-sensitivity C-reactive protein
