# Supplementary material for: Flap monitoring with incisional negative pressure wound therapy (NPWT) in diabetic foot patients
Source: Sci Rep. 2022 Sep 20;12:15684. doi: 10.1038/s41598-022-20088-9 (PMC9489718; doi:10.1038/s41598-022-20088-9)
Supplement: Supplementary file 1 — Supplementary Information. [file 41598_2022_20088_MOESM1_ESM.docx]

| Patients | Gender | Age | Comorbidity  (HTN, CVD) | Method  (Conventional/NPWT) | Flap  (Free/Perforator) | Result |
| --- | --- | --- | --- | --- | --- | --- |
| 1 | M | 53 | HTN, CVD | NPWT | Free | Success |
| 2 | M | 45 | - | Conventional | Perforator | Success |
| 3 | F | 84 | - | NPWT | Perforator | Success |
| 4 | M | 60 | - | NPWT | Perforator | Success |
| 5 | F | 55 | CVD | Conventional | Free | Success |
| 6 | F | 49 | HTN | Conventional | Free | Success |
| 7 | M | 61 | - | Conventional | Perforator | Success |
| 8 | M | 57 | HTN | NPWT | Free | Fail |
| 9 | M | 53 | HTN | Conventional | Perforator | Success |
| 10 | F | 66 | HTN, CVD | Conventional | Free | Success |
| 11 | F | 74 | - | NPWT | Free | Success |
| 12 | M | 47 | - | Conventional | Perforator | Success |
| 13 | F | 29 | CVD | Conventional | Perforator | Success |
| 14 | M | 61 | - | NPWT | Perforator | Success |
| 15 | M | 44 | HTN | NPWT | Perforator | Success |
| 16 | F | 68 | CVD | Conventional | Perforator | Success |
| 17 | M | 53 | - | NPWT | Free | Success |
| 18 | M | 54 | HTN | Conventional | Free | Success |
| 19 | F | 72 | - | NPWT | Free | Success |
| 20 | M | 51 | HTN | NPWT | Perforator | Success |
| 21 | F | 88 | - | Conventional | Perforator | Success |
| 22 | M | 50 | - | NPWT | Free | Success |
| 23 | M | 63 | HTN, CVD | NPWT | Perforator | Success |
| 24 | F | 55 | - | NPWT | Perforator | Success |
| 25 | M | 43 | CVD | NPWT | Perforator | Success |
| 26 | M | 59 | HTN | Conventional | Free | Fail |
